# Delayed Occlusal Loading of a Definitive Cutback Zirconia Crown in Immediate Implant Placement for Single-Tooth Replacement: A Case Report

**DOI:** 10.3390/jcm14228053

**Published:** 2025-11-13

**Authors:** Vincenzo Cosello, Andrea Parpaiola, Marco Toia

**Affiliations:** 1 Private Practice, 17011 Albissola Superiore, Italy; vinzcosello@gmail.com; 2 Department Clinic-Surgical Diagnostic and Pediatric Sciences, University of Pavia, 27100 Pavia, Italy; parpaiolaandrea@gmail.com; 3 Department of Oral and Maxillofacial Surgery and Oral Medicine, Faculty of Odontology, Malmö University, 20506 Malmö, Sweden

**Keywords:** immediate implant placement, guided surgery, one-time abutment

## Abstract

**Background/Objectives:** Immediate implant placement in the esthetic zone can shorten treatment time but maintaining peri-implant soft tissue stability is challenging. Conventional multi-stage workflows require multiple visits and may disturb peri-implant tissues. Placing a definitive one-time abutment at surgery can preserve soft tissue contours by avoiding multiple abutment changes. This case report introduces a digital one-stage approach delivering a definitive zirconia crown with delayed occlusal veneering at surgery to streamline treatment and preserve tissue stability. **Methods:** A 60-year-old male with a failing maxillary canine underwent immediate implant placement using guided surgery. A customized healing abutment preserved the emergence profile for the definitive restoration. A zirconia crown with an occlusal cut-back was fabricated and delivered at surgery on the one-time abutment without occlusal contact. After 12 weeks, a ceramic overlay was bonded extraorally to the crown to restore the occlusal surface. **Results:** At 2-year follow-up, the implant exhibited stable bone and healthy peri-implant soft tissues, with no complications. The one-time approach preserved tissue contours by eliminating provisional stages, and delayed occlusal veneering provided excellent esthetic integration. The patient was satisfied with the immediate result and fewer visits. This one-stage approach required fewer interventions than conventional provisional workflows. **Conclusions:** Immediate implant placement with a one-time abutment and delayed occlusal loading preserved peri-implant tissue architecture and achieved excellent functional and esthetic outcomes at 2 years. This one-stage workflow is a tissue-preserving alternative to multi-stage protocols; further studies are needed to confirm its long-term efficacy.

## 1. Introduction

The Immediate implant placement in the aesthetic zone represents an established treatment approach when conducted under optimal anatomical and clinical conditions. It offers key benefits, reduction in total treatment time, and improved patient satisfaction [1]. However, long-term success depends on maintaining the stability and volume of peri-implant soft tissues. Traditional multistage protocols often require five to six visits before the final prosthetic delivery [2]. This approach increases biological risk, chair time, and operational costs for the practice [3].

One of the most proposed technique is delivering the definitive abutment at the time of implant surgery and avoiding its removal during subsequent stages [4]. This strategy helps maintain soft tissue stability, protects the architecture of the biological width and promote an immediate stabilization of the supracrestal complex [5].

To the best of our knowledge, one-time abutments generally feature a standard design and lack anatomical customization relative to the specific tooth. Furthermore, customized abutments are most often limited to healing components or are produced through indirect workflows, which still involve multiple disconnections and conventional impression steps [6].

Advances in digital planning and ceramic materials now allow clinicians to adopt streamlined workflows that respect biological principles while improving efficiency [7].

One such strategy involves delivering a definitive monolithic zirconia crown prepared for delayed occlusal veneering on the same day as implant placement. This case report introduces a digitally guided, one-stage protocol that reduces the number of visits, while preserving high esthetical standards and enhancing patient comfort and practice cost-efficiency.

## 2. Materials and Methods

### 2.1. Case Presentation

**Figure 1:**
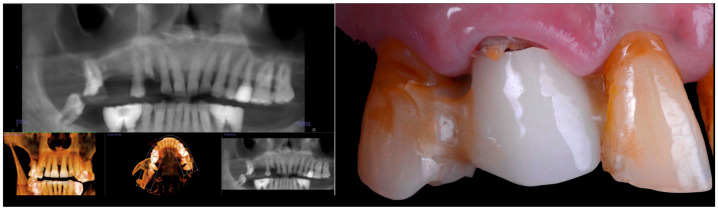
Radiographic and clinical image illustrating the extent of the carious lesion affecting the right maxillary canine.

A 60-year-old male patient with ASA 1 [8] presented with a non-restorable maxillary right canine (Figure 1).

The patient exhibited pre-existing soft tissue recession and generalized periodontal involvement, unrelated to the implant site. The periodontal condition was stabilized and remains under maintenance therapy. Due to socio-economic constraints, the patient opted to limit treatment to the replacement of the maxillary canine, while maintaining overall periodontal health through supportive care and follow-up visits.

The patient was offered two approaches: (i) or a bridge between the later and the premolar or (ii) an immediate implant placement following the canine extraction.

After the informed consent, preoperative assessment included cone beam computed tomography (KaVo OP 3D, Biberach an der Riß, Germany) and intraoral scanning (TRIOS 5, 3Shape A/S Niels Juels Gade 13, 1059 Copenhagen K, Denmark) were performed. Digital planning was completed using an implant planner (coDiagnostiX^®^, Institut Straumann AG, Basel, Switzerland), and a fully guided surgical protocol was established.

### 2.2. Surgery

**Figure 2:**
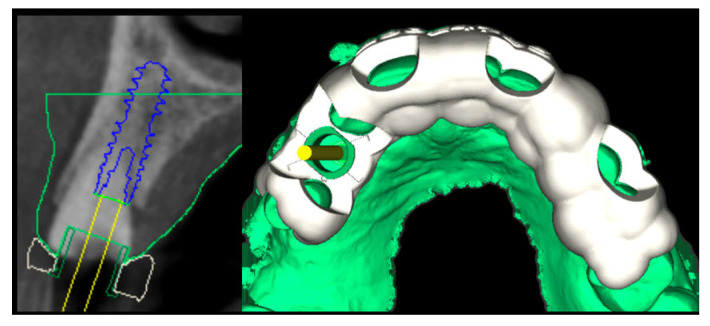
Planning of guided surgery with implant placement in the post-extractive site (**left side of the figure**) and the corresponding design of the surgical guide (**right side of the figure**).

**Figure 3:**
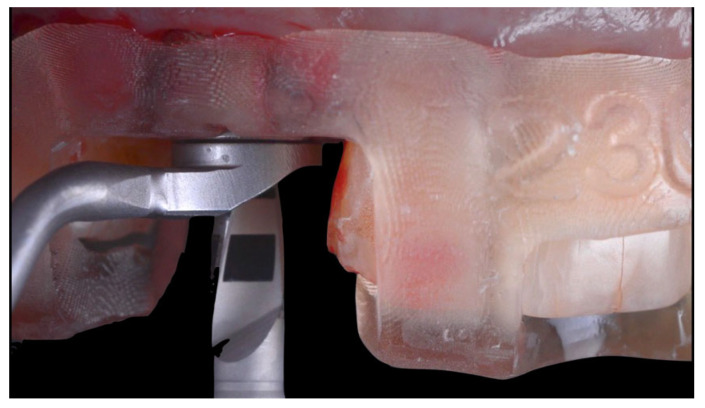
Clinical image illustrating implant placement using a static guided surgery approach.

Following atraumatic extraction, a conical connection implant (4.0 diameter/14 mm length, BLX, Institut Straumann AG, Basel, Switzerland) was placed guided (Figure 2 and Figure 3) for an immediate restoration. The implant was placed 1 mm sub-crestally into the fresh socket with a primary stability of 45 Ncm favorable and 4 mm from the gingival margin.

A connective tissue graft was applied buccally following a tunnel technique to enhance mucosal volume [9] and sutured (Pga 6.0 suture, Universal Sutures, Karnataka, India). Any bone substitute was inserted between the implant and the buccal bone plate since the jumping gap between the implant and the cortical plate was lower than 2 mm [10].

**Figure 4:**
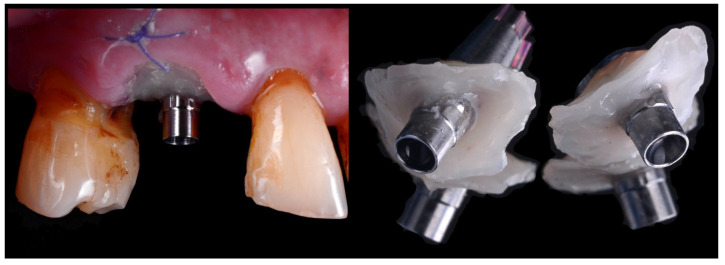
Images of two customized devices fabricated with flowable material on a titanium base to replicate the emergence profile within the supracrestal compartment. One device was sent to the laboratory for scanning and fabrication of the definitive crown, while the other was immediately placed as a healing abutment for several hours until definitive restoration.

Following the implant position two custom healing abutments were fabricated: one was sent to the laboratory to fabricate the Cutback Zirconia Crown scanning the emergence profile; the second as installed clinically as healing cup. To prepare these two specific customized devices, a titanium base (Variobase, Institut Straumann AG, Basel, Switzerland) was secured to the implant and realign with a flowable composite (Beautifil Flow, Shofu Dental Corporation, Kyoto, Japan) placed around the soft tissue to replicate the shape of the emergence profile of the supracrestal compartment [11]. Before applying the composite, a collagen sponge soaked with tranexamic acid was used to control bleeding and, at the same time, to protect the implant surface from contact with the composite material. After the light cured, one custom abutment was removed and sent to the laboratory to be scanned while the second was used to leave the patient two wait for the definitive crown (Figure 4). The implant position was detected using ta prefabricated scan body (Institut Straumann AG, Basel, Switzerland).

### 2.3. Laboratory Procedure

**Figure 5:**
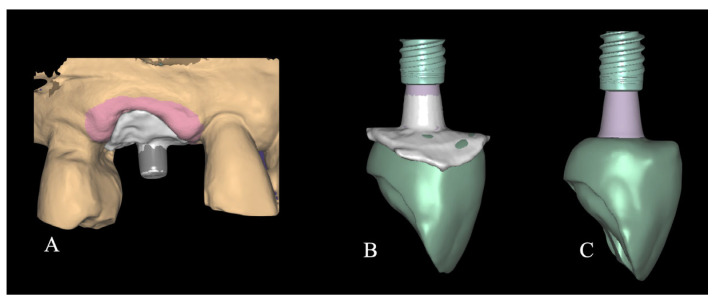
The figure illustrates the imported intraoral scan data showing the scanned titanium base in situ (**A**). It also displays the digital superimposition of the scanned titanium base with the anatomical contour, previously modeled in the patient’s mouth using a flowable composite (**B**). This superimposition accurately replicates the emergence profile and soft tissue interface, serving as a digital reference for the prosthetic design (**C**).

The laboratory procedure consisted in fabricating a monolithic zirconia crown with an occlusal cut-back design, bonded to a titanium base. The dental technician imported the intraoral scans, which were superimposed, and additionally, the scan of the customized titanium base in order to capture the soft tissue emergence profile. As a result, the emergence profile of the milled zirconia crown accurately reproduced the anatomical contour previously obtained using flowable composite (Figure 5).

A monolithic zirconia crown (Zirconia 3d pro multifunctional, 88dent company, Milano, Italy) was designed and intentionally milled with a non-functional activity in the occlusal surface, exhibiting infraocclusion contact. This strategy was adopted to minimize the risk of implant failure due to premature loading of a single-unit restoration [12].

**Figure 6:**
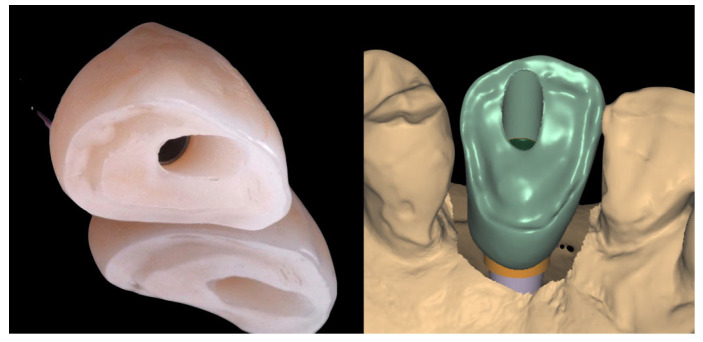
Occlusal cut-back area of the definitive zirconia framework, prepared with a thin layer of disilicate ceramic approximately 500 µm in thickness.

The occlusal cut-back area of the zirconia framework was prepared with a thin layer zone of disilicate ceramic (IPS e-max, Ivoclar Vivadent AG, Schaan, Liechtenstein) with a thickness of approximately 500 µm (Figure 6).

This procedure enabling the subsequent bonding of a definitive ceramic overlay after the complete osseointegration and soft tissue healing, approximately 12 weeks post-placement to allow for the final veneering step. This was performed using a chemical adhesive system to ensure optimal integration and long-term stability of the restoration. The crown was bonded to a titanium base using dual-cure resin cement (Panavia SE, Kuraray Noritake Dental Inc., Tokyo, Japan).

This procedure enabling the subsequent bonding of a definitive ceramic overlay after the complete osseointegration and soft tissue healing, approximately 12 weeks post-placement to allow for the final veneering step. This was performed using a chemical adhesive system to ensure optimal integration and long-term stability of the restoration. The crown was bonded to a titanium base using dual-cure resin cement (Panavia SE, Kuraray Noritake Dental Inc., Tokyo, Japan).

### 2.4. Crown Delivery

**Figure 7:**
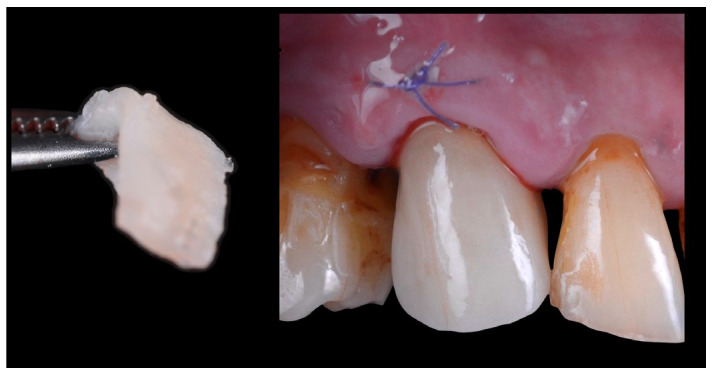
Definitive zirconia crown placed in situ, also showing the structure of the connective tissue graft inserted using the tunneling technique. On the left side of the image, the connective tissue is visible.

**Figure 8:**
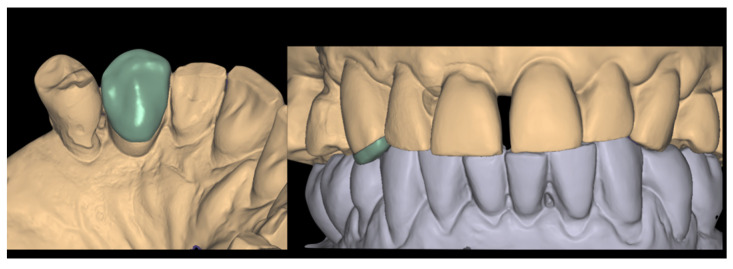
Image showing the scanned model in occlusion, highlighting the cutback area of the crown and the infra-occlusal contact region.

After 4 h from the surgical procedure, the customized healing abutment was removed and the definitive crown was secured and torqued according to the implant manufacturer’s guidelines (Figure 7 and Figure 8). The patient was instructed to rinse twice daily with 0.20% chlorhexidine for 15 days, maintain a liquid diet for 6 weeks, and avoid masticatory function in the treated area during this healing period. A lower occlusal splint was delivered to the patient in order to prevent any extrusion of the antagonistic teeth.

### 2.5. Definitive Overlay Adhesion

**Figure 9:**
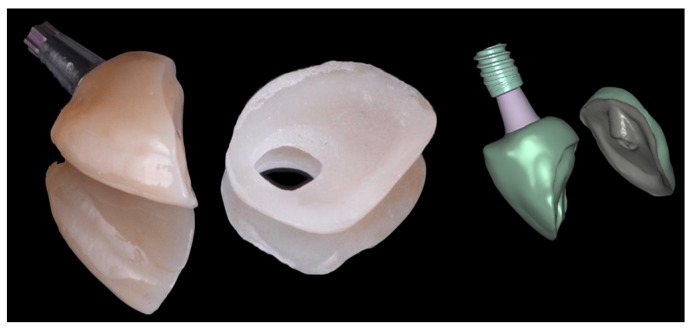
The Occlusal cut-back area of the definitive zirconia framework, prepared with a thin layer of disilicate ceramic approximately 500 µm in thickness and the ceramic overlay.

After 12 weeks, the definitive crown was removed, and the previously fabricated ceramic overlay (IPS e-max, Ivoclar Vivadent AG, Schaan, Liechtenstein) was bonded extraorally to the occlusal cut-back area of the zirconia framework prepared with a thin layer zone of disilicate ceramic. The bonding protocol followed standard adhesion procedures for ceramics [13]. The intaglio surface of the overlay as well as the ceramic layer of the crown was etched with hydrofluoric acid, followed by the application of phosphoric acid, silane coupling agent, and a universal adhesive (Figure 9).

**Figure 10:**
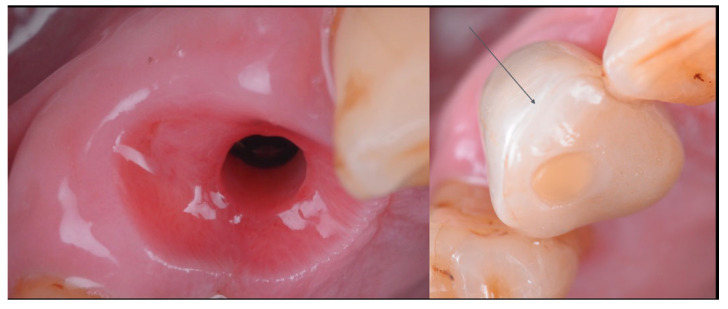
Healing of the peri-implant tissues after 12 weeks, prior to the placement of the definitive crown. On the right side of the figure, an image of the screw-retained crown. The arrow indicates the cementation border between the crown and the ceramic overlay. The crown was re-secured and torqued according to the manufacturer’s guidelines, and the screw access channel was sealed with composite resin.

Once the external surface of the restoration was polished, a gel containing sodium hypochlorite (Euclorina^®^, Dompé Farmaceutici SpA, Milan, Italy) was applied to the peri-implant soft tissues prior to re-insertion of the final restoration. The crown was re-secured and torqued according to the manufacturer’s guidelines. The screw access channel was sealed with composite resin (Figure 10).

**Figure 11:**
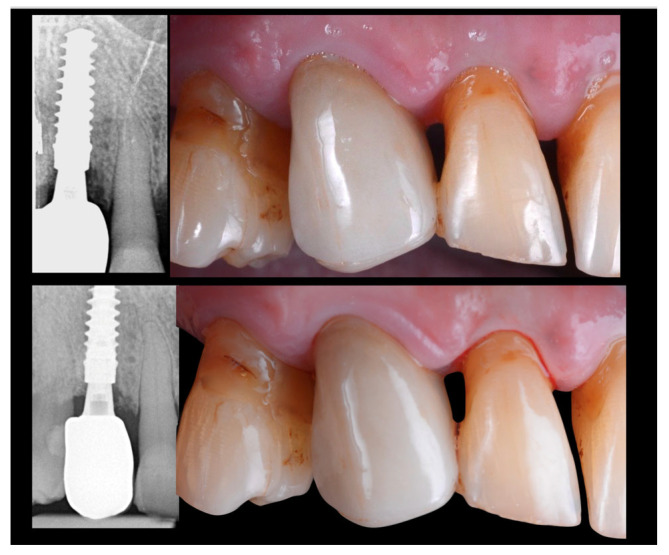
Clinical images and x-rays evaluation after the definitive crown installation (**upper side**) and after 2 years (**lower side**).

Final steps included occlusal adjustment, and the acquisition of a periapical radiograph to confirm the correct seating and integrity of the implant-supported restoration were applied. Clinical and radiological evaluation were assessed at 1 and 2 years (Figure 11).

## 3. Discussion

In the present case, the transmucosal contour was preserved by immediate implant placement combined with the simultaneous delivery of a custom-designed monolithic zirconia crown at the time of tooth extraction, using a fully guided surgical approach. The absence of any provisional restoration and of repeated abutment disconnections streamlined the procedure and minimized biological trauma during the initial healing phase. At 12 weeks post-surgery, the occlusal veneer was bonded in place, achieving excellent marginal adaptation and aesthetic integration. At 24 months, radiographic and clinical evaluations confirmed stable crestal bone levels and healthy peri-implant tissues. No technical or biological complications were observed throughout the follow-up period. The patient reported a high level of satisfaction, noting the immediate benefits of the treatment and a reduced number of clinical visits, which translated into less chair time and lower operational costs for the practice.

Post-extractive implant placement, particularly when coupled with guided surgery, represents a compelling modality in modern implant dentistry by offering numerous advantages over conventional approaches [12]. This technique aims to optimize both functional and aesthetic outcomes, streamlining the treatment process and enhancing patient comfort [14]. Achieving an ideal anatomical implant position is crucial for optimal restoration; however, this is not always feasible, as the physiological healing following tooth extraction, trauma, or pathology often leads to deficiencies in hard and soft tissues [15]. Guided surgery, leveraging advanced imaging and CAD/CAM technology, provides a predictable and precise method for implant placement, minimizing surgical invasiveness and maximizing utilization of available alveolar bone [16]. In scenarios requiring immediate loading, guided surgery facilitates the prefabrication of provisional restorations, enabling their immediate connection to the implant upon placement. This approach is particularly advantageous in cases demanding meticulous aesthetic control and immediate functional rehabilitation [17].

In the applied clinical protocol, provisional restorations were deliberately excluded in favor of the immediate placement of a definitive restoration. This restoration consisted of a zirconia framework fabricated to reproduce the natural tooth’s anatomical contours and was bonded to a titanium base. Such an approach avoided repeated abutment disconnections and reconnections, thereby safeguarding the continuity of the peri-implant soft tissue seal. Preserving this biological seal significantly lowered the likelihood of crestal bone remodeling and inflammatory reactions in the peri-implant area. Abrahamsson et al. [18] highlighted that repeated abutment manipulation may disrupt the establishment of the biological width, compromising the structural integrity of the peri-implant connective tissue. It has been proposed that continuous soft tissue disturbance exposes the supracrestal tissue compartment to bacterial infiltration, fostering inflammation and ultimately contributing to marginal bone loss [19].

Conversely, the placement of the definitive abutment at the time of surgery is thought to support the formation of a stable mucosal barrier. As shown in an in vivo investigation by Berglundh et al. [20] and further corroborated by clinical data from Tomasi et al. [21], soft tissue healing follows a progressive timeline: connective tissue fibers organize over 4 to 6 weeks, while complete epithelial coverage occurs between 6 and 8 weeks. Employing a definitive abutment from the day of implant placement enhances soft tissue maturation and limits marginal bone resorption compared to approaches involving multiple abutment changes [22]. Additional studies have confirmed that maintaining a consistent abutment connection can positively influence peri-implant tissue health [23]. Notably, in a recent 12-year multicenter study evaluating the effects of repeated abutment disconnections, Bressan et al. reported a reduced incidence of complications in implants that remained undisturbed throughout the healing phase [24].

In line with the principle of preserving soft tissue stability through the placement of the definitive abutment at the time of surgery, the present protocol is distinguished by its focus on the immediate and permanent definition of the transmucosal interface, without relying on interim restorations made of acrylic resin. Unlike conventional workflows, where the emergence profile is gradually shaped using provisional materials prone to surface degradation and repeated manipulation, in this case the tissue contour is established from the outset using a definitive material, capable of maintaining the morphology achieved during surgery unaltered.

This approach was executed using monolithic zirconia, a material recognized for its excellent biocompatibility, minimal bacterial adhesion, superior dimensional stability, and favorable interaction with peri-implant soft tissues. The emergence profile, shaped during surgery, was digitally captured and transferred to the laboratory by means of a second custom-designed abutment functioning as a reference guide. This technique ensured a precise replication of the biologically sculpted critical and subcritical contours in the definitive crown. The clinical reliability of this workflow is supported by recent studies: Chokaree et al. [25] demonstrated that customized healing abutments enhance soft tissue stability and aesthetic outcomes, while Ruhstorfer et al. [26] reported reduced marginal tissue loss and better preservation of the gingival architecture compared to conventional components.

The use of a monolithic zirconia crown further promoted peri-implant tissue stability. Zirconia has been shown to facilitate optimal mucosal integration and to retain less bacterial plaque than materials such as titanium or PMMA-based provisionals [27]. Its intrinsic biocompatibility and high surface smoothness help to reduce local inflammatory responses and may contribute to superior long-term aesthetic outcomes, especially in patients presenting with thin gingival biotypes. In the present case, the individualized emergence profile played a key role in establishing a natural and stable soft tissue architecture that remained unchanged during follow-up. The transmucosal interface maintained full continuity with the original surgical morphology, eliminating the need for postoperative modifications or abutment replacements. This stability fostered a sustained biological seal and effective tissue maturation, in line with evidence emphasizing the importance of preserving morphological integrity for long-term peri-implant health [28]. Within this context, the emergence profile functions not only as a prosthetic feature but also as an active biological component that contributes to soft tissue healing and preservation [29].

To complete the prosthetic procedure, the pre-fabricated ceramic overlay was bonded to the zirconia framework in accordance with validated conditioning protocols for glass-ceramics [30,31]. This protocol—combining micromechanical retention, chemical coupling, and high-performance adhesive cementation—has been shown to provide high bond strengths (>20–34 MPa) and long-term durability, even after thermal aging, as confirmed by systematic reviews and laboratory studies [32]. By separating the biological and adhesive phases and conducting bonding outside the oral cavity, the protocol ensured maximum procedural reliability while maintaining the morphological and biological integrity of the soft tissue interface. The protocol allowed for the use of a definitive abutment without occlusal load in the early healing phase of the implant, with loading being applied only after complete osseointegration had occurred. Case selection plays a key role in the success of this simplified workflow, as only patients presenting with adequate implant stability, preserved ridge morphology, and healthy peri-implant soft tissues can be considered suitable candidates for this type of restoration. Moreover, Occlusal conditions such as crossbite or reverse bite may affect esthetic integration and represent contraindications for this approach. Future studies with larger samples and long-term follow-up are required to confirm its predictability and broader clinical applicability.

## 4. Conclusions

This clinical case demonstrates that immediate implant placement with a digitally fabricated, monolithic zirconia crown can effectively preserve soft tissue contours, reduce surgical and prosthetic phases, and achieve excellent long-term aesthetic and functional outcomes.

- Immediate implant placement with a one-time abutment and delayed occlusal veneering preserved peri-implant soft tissue architecture.
- The protocol achieved excellent esthetic and functional outcomes at two-year follow-up.
- The one-stage workflow reduced surgical and prosthetic interventions while maintaining tissue stability.
- This approach may represent a predictable, tissue-preserving alternative to conventional multi-stage protocols.
- Further controlled studies are required to confirm long-term clinical reliability and reproducibility.

## Data Availability

No data is unavailable due to privacy.

## Abbreviations

The following abbreviations are used in this manuscript:

ASA	American Society of Anesthesiologists
